# The photosynthetic and structural differences between leaves and siliques of *Brassica napus* exposed to potassium deficiency

**DOI:** 10.1186/s12870-017-1201-5

**Published:** 2017-12-11

**Authors:** Zhifeng Lu, Yonghui Pan, Wenshi Hu, Rihuan Cong, Tao Ren, Shiwei Guo, Jianwei Lu

**Affiliations:** 1 Collge of Resources and Environment, Huazhong Agricultural University, Key Laboratory of Arable Land Conservation (Middle and Lower Reaches of Yangtze River) Ministry of Agriculture, Shizishan Street 1, Wuhan, 430070 People’s Republic of China; 2 Jiangsu Provincial Key Lab for Organic Solid Waste Utilization, National Engineering Research Center for Organic-based Fertilizers, Jiangsu Collaborative Innovation Center for Solid Organic Waste Resource Utilization, Nanjing Agricultural University, Nanjing, 210095 People’s Republic of China

**Keywords:** *Brassica napus* L., Leaf photosynthesis, Potassium deficiency, Silique photosynthesis, Structural properties

## Abstract

**Background:**

Most studies of photosynthesis in chlorenchymas under potassium (K) deficiency focus exclusively on leaves; however, little information is available on the physiological role of K on reproductive structures, which play a critical role in plant carbon gain. *Brassica napus* L., a natural organ-succession species, was used to compare the morphological, anatomical and photo-physiological differences between leaves and siliques exposed to K-deficiency.

**Results:**

Compared to leaves, siliques displayed considerably lower CO_2_ assimilation rates (*A*) under K-deficient (−K) or sufficient conditions (+K), limited by decreased stomatal conductance (*g*_s_), apparent quantum yield (α) and carboxylation efficiency (CE), as well as the ratio of the maximum rate of electron transport (*J*_max_) and the maximum rate of ribulose 1,5-bisphosphate (RuBP) carboxylation (*V*_cmax_). The estimated *J*_max_, *V*_cmax_ and α of siliques were considerably lower than the theoretical value calculated on the basis of a similar ratio between these parameters and chlorophyll concentration (i.e. *J*_max_/Chl, *V*_cmax_/Chl and α/Chl) to leaves, of which the gaps between estimated- and theoretical-*J*_max_ was the largest. In addition, the average ratio of *J*_max_ to *V*_cmax_ was 16.1% lower than that of leaves, indicating that the weakened electron transport was insufficient to meet the requirements for carbon assimilation. Siliques contained larger but fewer stoma, tightly packed cross-section with larger cells and fewer intercellular air spaces, fewer and smaller chloroplasts and thin grana lamellae, which might be linked to the reduction in light capture and CO_2_ diffusion. K-deficiency significantly decreased leaf and silique *A* under the combination of down-regulated stomatal size and *g*_s_, chloroplast number, α, *V*_cmax_ and *J*_max_, while the CO_2_ diffusion distance between chloroplast and cell wall (*D*_chl-cw_) was enhanced. Siliques were more sensitive than leaves to K-starvation, exhibiting smaller reductions in tissue K and parameters such as *g*_s_, *V*_cmax_, *J*_max_ and *D*_chl-cw_.

**Conclusion:**

Siliques had substantially smaller *A* than leaves, which was attributed to less efficient functioning of the photosynthetic apparatus, especially the integrated limitations of biochemical processes (*J*_max_ and *V*_cmax_) and α; however, siliques were slightly less sensitive to K deficiency.

**Electronic supplementary material:**

The online version of this article (10.1186/s12870-017-1201-5) contains supplementary material, which is available to authorized users.

## Background

Carbon assimilation by chlorenchymal tissues contributes more than 90% of crop biomass [1]. Among chlorenchymas, leaves have long been considered as the principal organ responsible for photosynthetic activity in vascular plants, and the net CO_2_ assimilation of leaves has been studied extensively. However, mounting evidence indicates that non-foliar organs, such as reproductive structures, green stems, petioles, peduncles and roots, contain well-developed chloroplasts and contribute substantially to net carbon assimilation [2–5]. Among them, reproductive organs, such as siliques, panicles and cotton bolls, are usually green during their early development and contribute to the resource pool; however, they gradually become sink during maturation and senescence [6]. Therefore, fruit CO_2_ assimilation may particularly important for those plants to acquire extra CO_2_ and assimilates storage. Potassium (K), which is the most abundant univalent cation in plants, plays a crucial role in facilitating photosynthesis, construction of reproductive organs and crop yield. Previous studies have demonstrated the critical role of K in leaf photosynthesis [7–10]; however, this role remains to be confirmed in non-foliar organs.

Photosynthetically active organs can be divided into two groups according to their carbon gain. One group is characterized by net carbon assimilation using mainly atmospheric CO_2_, and another group performs effective utilization of respiratory CO_2_ [11]. Leaves, usually in the form of blades, absorb CO_2_ from the atmosphere mainly through the lower epidermal stomata, and deliver it across the mesophyll layers to the sites of carboxylation. K deficiency is known to limit leaf photosynthesis through diffusion resistance and biochemical obstacles [12]. K-starvation considerably decreases leaf stomatal conductance (*g*_s_) and therefore, blocks the uptake of atmospheric CO_2_ leading to down-regulation of net photosynthetic rate (*A*) [13, 14]. The non-foliar organs also contain stomatal pores, which, however, are quite different from that of leaf, generally with bigger stoma but lower density [11]. Their carbon demands are met either from the atmosphere or by reusing internally recycled CO_2_, or both. Despite progress in our understanding of the influence of K on leaf stomatal aperture, the effect of K on the non-foliar stomatal traits is remains to be elucidated. Mesophyll conductance (*g*_m_) has long been considered a key factor, the influence of which is comparable to that of *g*_s_ in determining leaf CO_2_ diffusion [15]. Furthermore, *g*_m_ is down-regulated under K-deficiency as a consequence of the decreasing chloroplast surface area exposed to the airspace and increasing cytoplasmic resistance [10]. However, mesophyll conductance in non-leaf organ remains to be investigated. CO_2_ assimilation in chloroplast requires energy consumption that is dependent on Rubisco (ribulose 1,5-bisphosphate carboxylase) carboxylation. K-deficiency accelerates the degradation of leaf chloroplasts, resulting in chlorosis which reduces energy capture as well as the rate of electron transport and carboxylation [8, 13, 14, 16]. Non-leaf organs contain well-developed chloroplasts; however, their chlorophyll content is only between 15% and 33% of that in the respective leaves [11, 17]. This may affect the absorption and utilization of light energy, as well as the electron transport process and carboxylation rate. Additionally, swollen, or even ruptured chloroplasts, with poor contrast and obscure grana stacks are occasionally observed in K-starved leaves [18]. Since the integrity of the thylakoid membrane is essential for leaf CO_2_ assimilation, the down-regulation of *A* under K-deficient conditions may be partly ascribed to the decrease in photochemical efficiency [12, 19]. In contrast, little information is available on the structural variation of chloroplasts in non-foliar organs under K-starvation. Overall, the evidences described here suggests that there are differences in photosynthesis between leaves and non-foliar organs, with K levels presumably influencing organ photosynthetic capacity through structural and physiological regulation.

Winter oilseed rape (*Brassica napus* L.), an herbaceous annual plant, presents an obvious succession of photosynthetic organs during the process of growth (Additional file 1: Figure S1). Leaves, as the most important photosynthetic structure before the flowering stage, are responsible for generating and deploying carbohydrates in the construction of plant architecture and silique walls. At the onset of flowering, the decline in the leaf area index (LAI) is accelerated as a result of shading by the canopy, initially comprising yellow flowers and later, the siliques [20, 21]. At the same time, silique area increases rapidly and, peaks at the start of ripening, with a maximum pod area index (PAI) equal to, or slightly less than, the LAI [21]. Specifically, leaves are the main photosynthetic structure before flowering stage, however, they are gradually senescent and separate from the plant beginning from the onset of flowering. Meanwhile, the siliques start to growth and occupy the hole canopy, and ultimately replace leaf as predominant carbon gain organs. The silique canopy intercepts approximately 80% of the incident radiation, and contributes to 80 to 95% of the total carbon gain during the pod filling stage [22]. Taken together, leaves and siliques are the two most important photosynthetic organs during the entire period of rapeseed growth. K-deficiency, in combination with a functional decline in leaf photosynthesis, causes rapeseed yield loss [23]. However, previous studies focusing on the influence of K in siliques, especially in CO_2_ assimilation, are rare. Therefore, in this study, *Brassica napus* L. was selected as a model plant to evaluate the differences between leaves and siliques and, their response to K-starvation. The aims of the current study were: (1) to compare the anatomical and photosynthetic differences between leaves and siliques by combining anatomical techniques with, gas exchange and chlorophyll fluorescence analyses; (2) to clarify the photosynthetic response of siliques under K-deficiency and the possible mechanism.

## Results

### Morpho-physiological traits of leaves and siliques

**Fig. 1:**
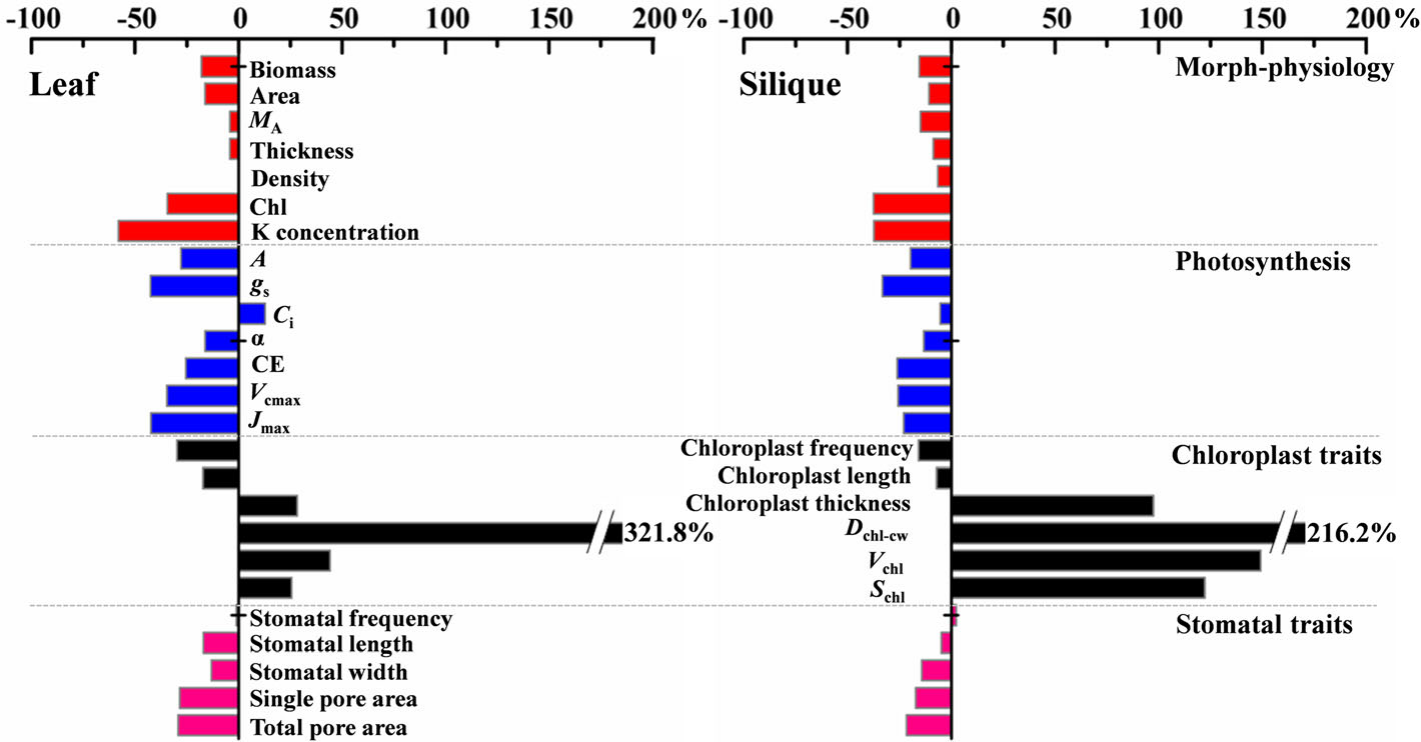
Changes in morph-physiological (red bars), photosynthetic (blue bars), stomatal (black bars) and chloroplastic traits (pink bars) of leaves and siliques in response to K-deficiency. The response ratio was calculated as the relative variation of each parameters under K-deficiency using the +K treatment as a control. Value bars facing the left and right indicate decreases and increases in traits compared to the values under +K treatment, respectively. *M*_A_, leaf (silique) mass per area; Chl, chlorophyll content; *A*, net photosynthetic rate; *g*_s_, stomatal conductance; *C*_i_, substomatal CO_2_ concentration; α, apparent quantum yield; CE, carboxylation efficiency; *V*_cmax_, the maximum rate of RuBP carboxylation; *J*_max_, the maximum rate of electron transport. *D*_chl-cw_, the distance between chloroplast and cell wall; *V*_chl_, chloroplast volume; *S*_chl_, chloroplast surface area

The biomass, area and chlorophyll concentration of individual leaves were significantly higher than the corresponding indexes of siliques, whereas siliques showed superiority in density and K concentration (Table 1). K-deficiency profoundly limited the growth of leaves and siliques, resulting in a reduction in most of the studied morpho-physiological traits (except that density was independent of K nutrition). Specifically, biomass was the most affected index under K-deficiency among all the morphological traits, with a 17.9% and 15.4% decrease in leaves and siliques, respectively (Table 1 and Fig. 1). In comparison with +K treatment, the chlorophyll concentration of leaves and siliques decreased by approximately 35.0%, with a more marked decline in K concentration in leaves compared with that in silique (Table 1 and Fig. 1).

**Table 1:** Effects of K supply on morphological and physiological traits of leaves and siliques

Organs	Treatment	Biomass (g)	Area (cm^2^)	*M*_A_ (g m^−2^)	Thickness (μm)	Density (g cm^−3^)	Chl (g m^−2^)	K concentration (%)
Leaf	-K	2.48 ± 0.17b	336.8 ± 28.2b	73.9 ± 1.8a	313.9 ± 4.3b	0.235 ± 0.006a	0.42 ± 0.06b	0.89 ± 0.06b
	+K	3.02 ± 0.07a	401.9 ± 14.7a	77.1 ± 2.3a	327.6 ± 2.8a	0.235 ± 0.007a	0.64 ± 0.06a	2.12 ± 0.05a
Silique	-K	0.11 ± 0.00b*	6.6 ± 0.1b*	73.1 ± 3.8b	304.8 ± 5.2b*	0.240 ± 0.012a	0.20 ± 0.01b*	2.05 ± 0.06b*
	+K	0.13 ± 0.01a*	7.4 ± 0.2a*	85.9 ± 2.0a*	333.6 ± 4.8a	0.257 ± 0.006a*	0.32 ± 0.02a*	3.27 ± 0.03a*

*M*_A_, leaf (silique) mass per area; Chl, chlorophyll content. Data represent mean ± standard error (SE) of four replicates for biomass, area, *M*_A_, density, Chl and K concentration, and at least 16 replicates for leaf thickness

Different letters indicate statistically significant differences (*P* < 0.05) between the -K and +K treatments

*Indicates statistically significant differences (*P* < 0.05) between the two organs under the same treatment conditions

### Response of photosynthesis to irradiation and CO_2_ concentration

Under light saturation conditions, the net photosynthetic rate (*A*) of leaves under the -K and +K treatments were 2.6 and 2.9 times the rates of siliques, respectively (Table 2). Leaf stomatal conductance (*g*_s_) was also considerably higher than that of siliques; however, intercellular CO_2_ concentration (*C*_i_) was slightly lower in leaves. K-deficiency significantly down-regulated *A* and *g*_s_, yet had completely opposite effects on *C*_i_ between leaves and siliques. In terms of *A* and *g*_s_, leaves were more sensitive than siliques to K deficiency (Fig. 1).

**Table 2:** Effects of K supply on photosynthetic parameters of light- and CO_2_-response curves

Organs	Treatment	*A* (μmol m^−2^ s^−1^)	*g*_s_ (mol m^−2^ s^−1^)	*C*_i_ (μmol mol^−1^)	α	α/Chl (m^−2^/g)	CE	CE/Chl (m^−2^/g)
Leaf	-K	16.8 ± 1.7b^1^	0.199 ± 0.009b	264 ± 7a	0.0463 ± 0.0023b	0.1103 ± 0.0054a	0.0604 ± 0.0012b	0.1438 ± 0.0027a
	+K	23.3 ± 1.1a	0.346 ± 0.012a	234 ± 6b	0.0552 ± 0.0022a	0.0863 ± 0.0034b	0.0811 ± 0.0008a	0.1267 ± 0.0022b
Silique	-K	6.5 ± 0.1b*	0.108 ± 0.009b*	281 ± 4a	0.0184 ± 0.0004b*	0.0921 ± 0.0018a*	0.0304 ± 0.0003b*	0.1521 ± 0.0028a
	+K	8.1 ± 0.3a*^2^	0.162 ± 0.004a*	297 ± 4a*	0.0212 ± 0.0003a*	0.0663 ± 0.0009b*	0.0411 ± 0.0002a*	0.1285 ± 0.0008b

*A*, net photosynthetic rate; *g*_s_, stomatal conductance; *C*_i_, substomatal CO_2_ concentration; α, apparent quantum yield; α/Chl, apparent quantum yield per chlorophyll concentration; CE, carboxylation efficiency; CE/Chl, carboxylation efficiency per chlorophyll concentration

Data represent mean ± standard error (SE) of four replicates for parameters under light saturation conditions and three replicates for simulation parameters of light- and CO_2_-response curves

Different letters indicate statistically significant differences (*P* < 0.05) between the -K and +K treatments

*Indicates statistically significant differences (*P* < 0.05) between the two organs under the same treatment conditions

**Fig. 2:**
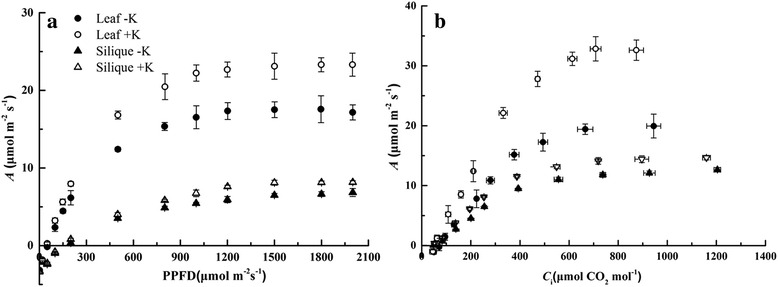
Light (**a**) and CO_2_ (**b**) response curves showing the net photosynthetic rate (*A*) in leaves and siliques under K-deficiency (−K) and sufficient K supply (+K) treatments. Values represent mean ± standard error (SE) of three replicates

As irradiations and CO_2_ concentrations increased, *A* increased rapidly, peaked and finally stabilized (Fig. 2). It could be concluded from the simulation parameters of the light- and CO_2_-response curves that the apparent quantum yield (α) and carboxylation rate (CE) were enhanced in leaves compared with the values in siliques (Table 2). The ratio between α and chlorophyll concentration (α/Chl) was higher in leaves versus that of siliques; however, there was no difference in the ratio of CE to chlorophyll concentration (CE/Chl) between leaves and siliques. K-deficiency significantly decreased the initial rate of increase and maximum values of *A*, α/Chl and CE/Chl. The response of leaves to K-deficiency was slightly higher than that of siliques (Fig. 1).

### Mapping chlorophyll fluorescence

**Fig. 3:**
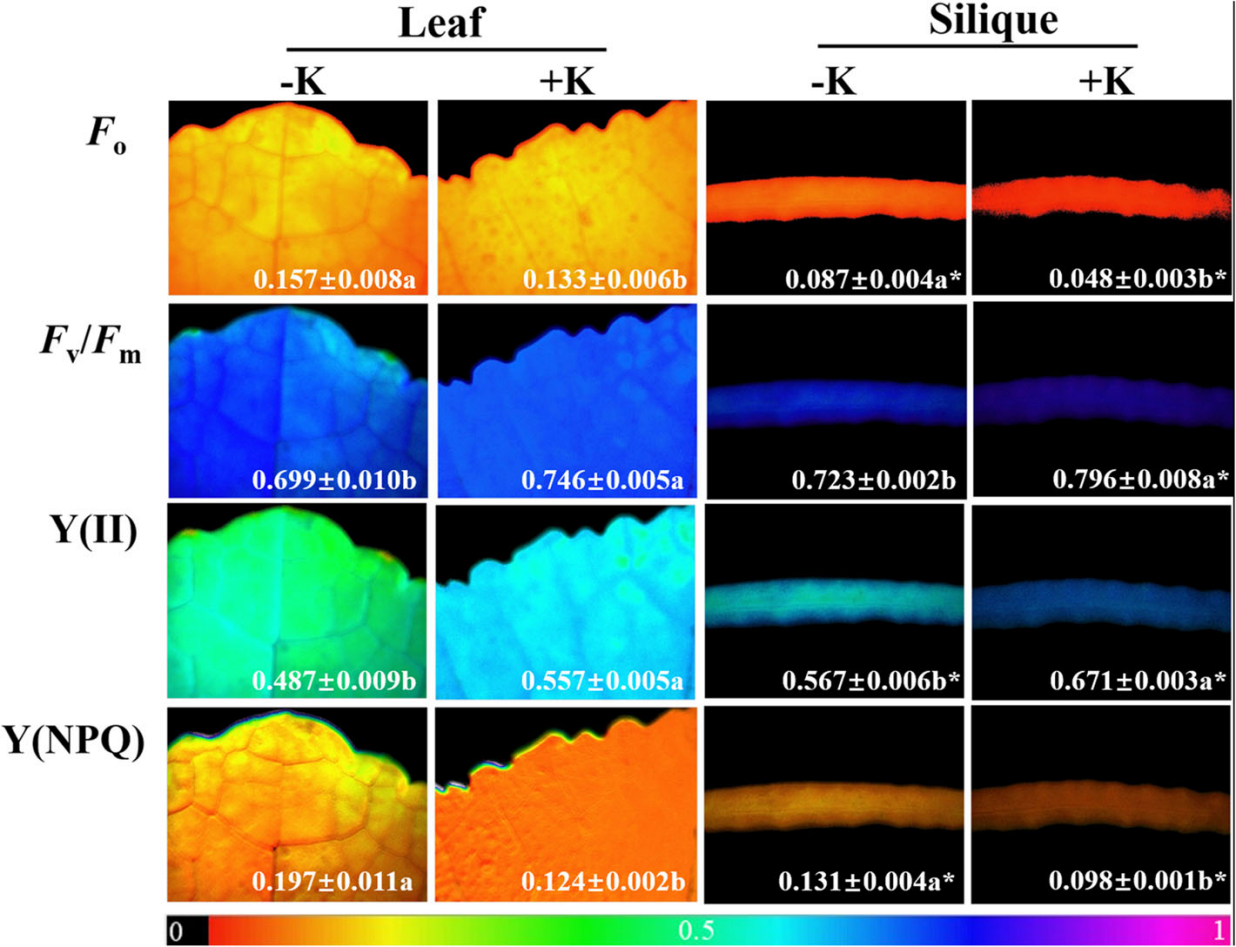
Images obtained by imaging-PAM analysis of leaves and siliques in K-deficiency (−K) and sufficient K supply (+K) treatments. Values represent mean ± standard error (SE) of three replicates indicate the intensity of each parameter. *F*_o_: minimum fluorescence; *F*_v_/*F*_m_: maximum quantum yield of PSII; Y(II), actual photochemical efficiency of PSII; Y(NPQ), quantum yield of regulated energy dissipation

The imaging-PAM analysis showed weaker minimum fluorescence (*F*_o_) and quantum yield of regulated energy dissipation (Y(NPQ)) in siliques compared with leaves; however, the maximum quantum yield of PSII (*F*_v_/*F*_m_) and actual photochemical efficiency of PSII (Y(II)) was higher (Fig. 3). The average *F*_o_ values of the organs evaluated were significantly higher under K-deficiency than those in the +K treatment groups. Compared with the -K treatment, +K treatment improved Y(II) by 14.4% and 18.3% in leaves and siliques, respectively; nevertheless, for Y(NPQ), decreases of 37.1% and 25.2%, respectively, were observed. Furthermore, K supply improved *F*_v_/*F*_m_ of leaves and siliques. Obvious heterogeneities were observed in the images, especially the maps captured from leaves in the -K treatment, with enhanced Y(NPQ) and reduced Y(II).

### Anatomical traits of leaves and siliques

**Fig. 4:**
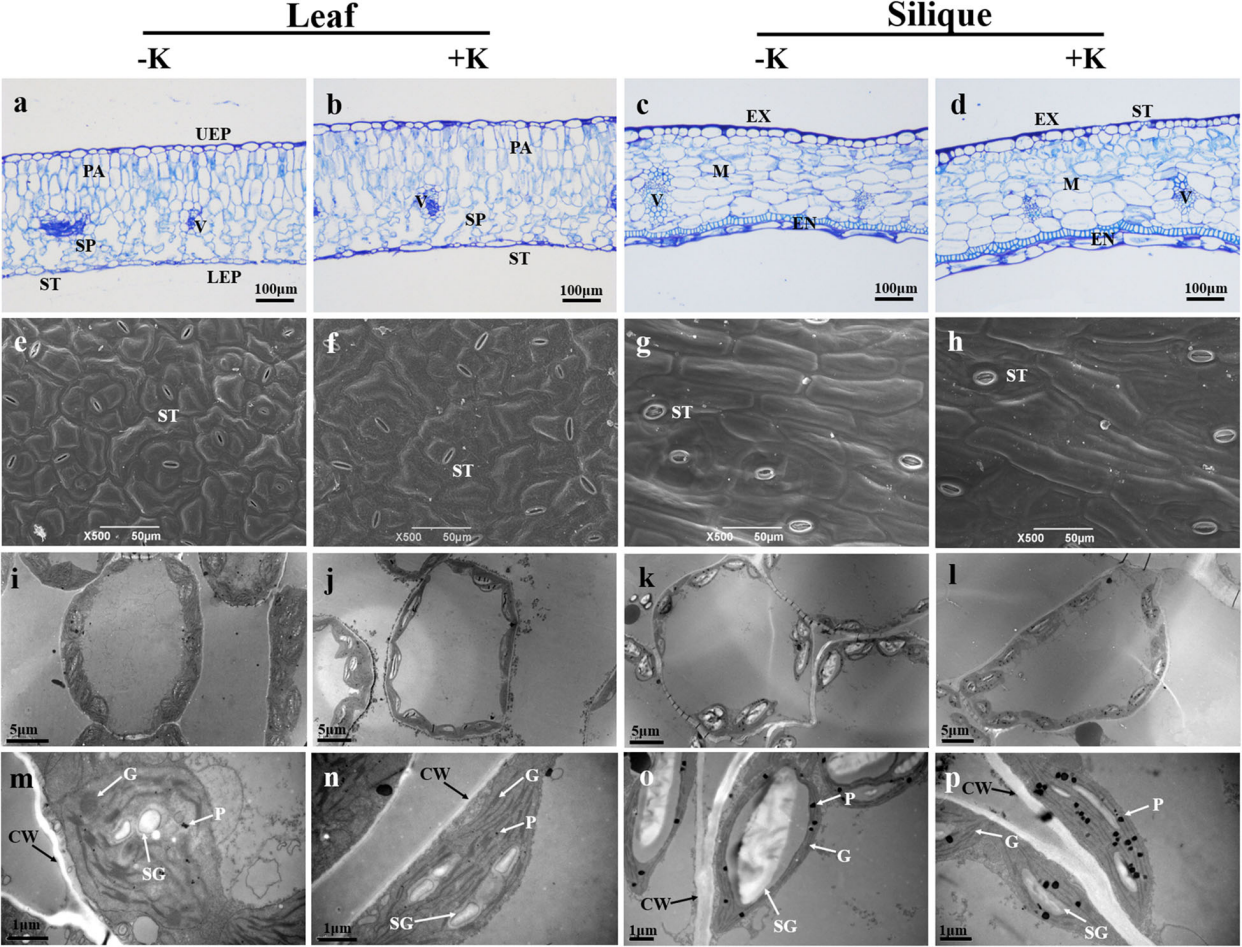
Representative light micrographs (**a**, **b**, **c** and **d**; scale bar = 100 μm), scanning electron micrographs (**e**, **f**, **g** and **h**; scale bar = 50 μm) and transmission electron micrographs (**i**, **j**, **k** and **l**; scale bar = 5 μm; **m**, **n**, **o** and **p**; scale bar = 1 μm) of leaves and siliques under K-deficiency (−K) and sufficient K supply (+K) treatments. UEP, upper epidermis; LEP, lower epidermis; PA, palisade layers; SP, spongy layers; V, vascular bundle; ST, stomata; EX, exocarp; M, mesocarp; EN, endocarp; CW, cell wall; G, granum; P, plastoglobuli; SG, starch grain

The leaf and pericarp structures showed marked differences. Leaves consisted of two epidermal layers (upper and lower), palisade and spongy layers (Fig. 4a, b), while the pericarp classified into three functional layers-the exocarp, mesocarp and endocarp (Fig. 4c, d). The leaf epidermis and exocarp were formed by single-celled epidermal layers, while the palisade, spongy layers (Fig. 4a, b) and mesocarp were composed of layers of chlorenchyma cells, and the endocarp consisted of large thin-walled cells and an inner layer with small, tightly packed cells (Fig. 4c, d). There was a large intercellular air space in the spongy layers of leaves, but not in the mesocarp layer of the pericarp. Additionally, K supply significantly improved the thickness of leaves and siliques (Table 1 and Fig. 4a-d).

Stomatas were distributed in the leaf epidermis and silique exocarp, and the average number of stomata in the lower leaf epidermis was approximately 5-fold higher than that of the silique outer epidermis; however, the silique stomata size, i.e., stomatal length, width and single pore area, were much larger (Table 3 and Fig. 4e-h). The total pore area, which is the product of stomatal frequency and single pore area, was considerably increased in leaves. K deficiency decreased stomatal size, but had no influence on stomatal frequency.

**Table 3:** Effects of K-deficiency on stomatal characteristics of leaves and siliques

Organs	Treatment	Frequency (no. mm^−2^)	Length (μm)	Width (μm)	Single pore area (μm^2^)	Total pore area (μm^2^)
Leaf	-K	348.5 ± 7.5a^1^	8.51 ± 0.21b	3.10 ± 0.12b	21.19 ± 1.12b	7.39 ± 0.44b
	+K	352.6 ± 10.8a	10.26 ± 0.26a	3.57 ± 0.12a	29.58 ± 0.71a	10.43 ± 0.57a
Silique	-K	72.79 ± 4.76a*^2^	13.39 ± 0.30b*	6.40 ± 0.25b*	65.15 ± 3.43b*	4.39 ± 0.19b*
	+K	71.07 ± 2.21a*	14.05 ± 0.20a*	7.47 ± 0.24a*	78.73 ± 2.43a*	5.60 ± 0.17a*

Data represent mean ± standard error (SE) of at least 20 replicates for stomatal frequency and 50 replicates for other traits

Different letters indicate statistically significant differences (*P* < 0.05) between the -K and +K treatments

*Indicates statistically significant differences (*P* < 0.05) between two organs under the same treatment conditions

Compared with siliques, leaf mesophyll cells contained more chloroplasts, which were greater in length and thickness, as well as enlarged chloroplast surface area and volume (Table 4 and Fig. 4m-p). Occasional starch granules were observed in the leaf chloroplasts (Fig. 4i, j); however, they were ubiquitously present in the chloroplasts of silique cells (Fig. 4k, l). In addition, the grana lamellae were thicker in leaf chloroplasts. The chloroplast frequency per cell decreased in the -K treatment, nevertheless, the chloroplast size and the distance between the chloroplast and cell wall were significantly enhanced. In the presence of a sufficient K supply, chloroplasts were regular ellipsoidal in shapes, the granum thylakoid was well-developed and the grana lamellae structures were clear and integral (Fig. 4n, p). However, in K-starved leaves, the chloroplast envelope was swollen and even ruptured in some cases, with a circular profile, and the granum thylakoid was irregularly arranged (Fig. 4m). In silique chloroplasts, K-deficiency caused marked starch accumulation, which ultimately resulted in a 2.2-fold increase in volume compared with that observed in the +K treatment (Table 4 and Fig. 4o). The average distances between the chloroplast and cell wall (*D*_chl-cw_) of leaves and siliques were markedly enhanced in the -K treatment, with a greater distance between chloroplasts and the cell wall in leaves (Table 4 and Fig. 1).

**Table 4:** Effects of K-deficiency on chloroplast ultrastructure of leaves and siliques

Organs	Treatment	Frequency (no. per cell)	Length (μm)	Thickness (μm)	*D*_chl-cw_ (μm)	*V*_chl_ (μm^3^)	*S*_chl_ (μm^2^)
Leaf	-K	10.9 ± 0.9a^1^	5.77 ± 0.20b	3.13 ± 0.13a	0.426 ± 0.057a	32.03 ± 2.69a	47.18 ± 2.77a
	+K	15.5 ± 1.2a	6.97 ± 0.18a	2.44 ± 0.08b	0.101 ± 0.010b	22.21 ± 1.44b	37.57 ± 1.60b
Silique	-K	11.2 ± 0.7b	4.96 ± 0.80a*	3.06 ± 0.89a	0.313 ± 0.021a*	26.48 ± 3.43a*	41.58 ± 3.50a*
	+K	13.3 ± 1.2a*^2^	5.34 ± 1.46a*	1.55 ± 0.62b*	0.099 ± 0.005b	10.63 ± 2.32b*	18.71 ± 2.92b*

Data represent mean ± standard error (SE) of at least 30 replicates. *D*_chl-cw_, the distance between the chloroplast and cell wall; *V*_chl_, chloroplast volume; *S*_chl_, chloroplast surface area

Different letters indicate statistically significant differences (*P* < 0.05) between the -K and +K treatments

*Indicates statistically significant differences (*P* < 0.05) between the two organs under the same treatment conditions

## Discussion

### The enhanced gap in the average *A* between leaves and siliques is associated with integrated limitations of biochemical processes (*J*_max_ and *V*_cmax_) and apparent quantum yield (α)

**Fig. 5:**
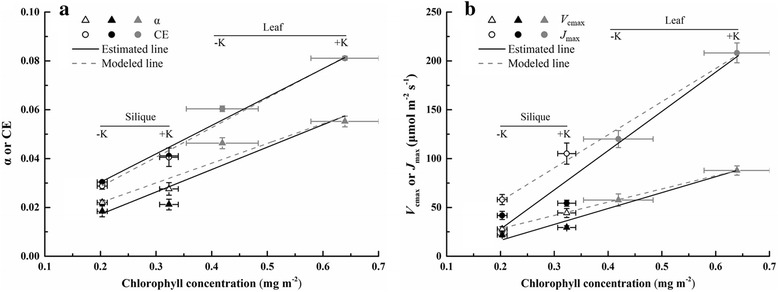
The relationship between estimated and modeled (**a**) apparent quantum yield (α) and carboxylation efficiency (CE), (**b**) the maximum rate of electron transport (*J*_max_) and the maximum rate of RuBP carboxylation (*V*_cmax_) of siliques with chlorophyll concentrations. The dashed gray lines in (**a**) represent the modeled α or CE, assuming that the ratio between α or CE and chlorophyll concentration of siliques was the same as the values for leaves, and indicate the modeled *J*_max_ or *V*_cmax_ under the assumption that the ratio between *J*_max_ or *V*_cmax_ and chlorophyll concentration of siliques was the same as the values for leaves in (**b**). The solid black lines denote the values estimated from the light and CO_2_ response curves. Values represent mean ± standard error (SE) of three replicates

The silique wall has been asserted as a modified leaf [6, 17], optimized for plant light harvesting and yield performance. The average net photosynthetic rate (*A*) of silique wall was 6.5 to 8.1 μmol m^−2^ s^−1^ depending on different K treatments, with the range of values commonly reported [22]. Silique *A* was only approximately 35.0% that of leaf, analogous to many non-foliar organs for which *A* is between 20 and 80% of that in leaves [4, 11]. The rate of photosynthesis is dependent on energy and reductant availability and the biochemical synthesis of carbohydrates with CO_2_ as a substrate. In the present study, these factors were evaluated based on the combination of light- and CO_2_-response curves and chlorophyll fluorescence. Leaf *A* was much higher than that of siliques, regardless of the gradients of light intensity or CO_2_ concentration (Fig. 2). Similar relationships have been reported for the comparison of leaf- and non-leaf photosynthesis in *Zantedeschia aethiopica* [24] and *Helleborus viridis* [4]. The two chlorophyll-related parameters, apparent quantum yield (α) and carboxylation efficiency (CE), which can be modeled from the initial slope of the linear part of light- and CO_2_-response curves, played important roles in regulating *A* by influencing light harvesting and ribulose 1,5-bisphosphate (RuBP) carboxylation. Leaves showed discernible advantages in α and CE; however, when based on their own chlorophyll concentration, leaf α/Chl was slightly higher than that of siliques, while the CE/Chl ratios of the two organs were similar (Table 2). If silique α/Chl and CE/Chl were assumed to be the same as those of leaves, the CE values estimated from the *A*-*C*_i_ curves were close to the modeled values (Fig. 5a). Nevertheless, the estimated α values were 16.6% and 23.2% lower than the theoretical values in the -K and +K treatment, respectively (Fig. 5a and Additional file 2: Table S1). For this reason, α was deemed to be more important than CE in determining the difference in *A* between leaves and siliques. However, the opposite result was reported for *Zantedeschia aethiopica* in that the CE of petioles decreased by 65.8% in compared with the theoretical value under the assumption of consistent CE/Chl between leaves and petioles; however, the variation in α was less pronounced [4].

**Fig. 6:**
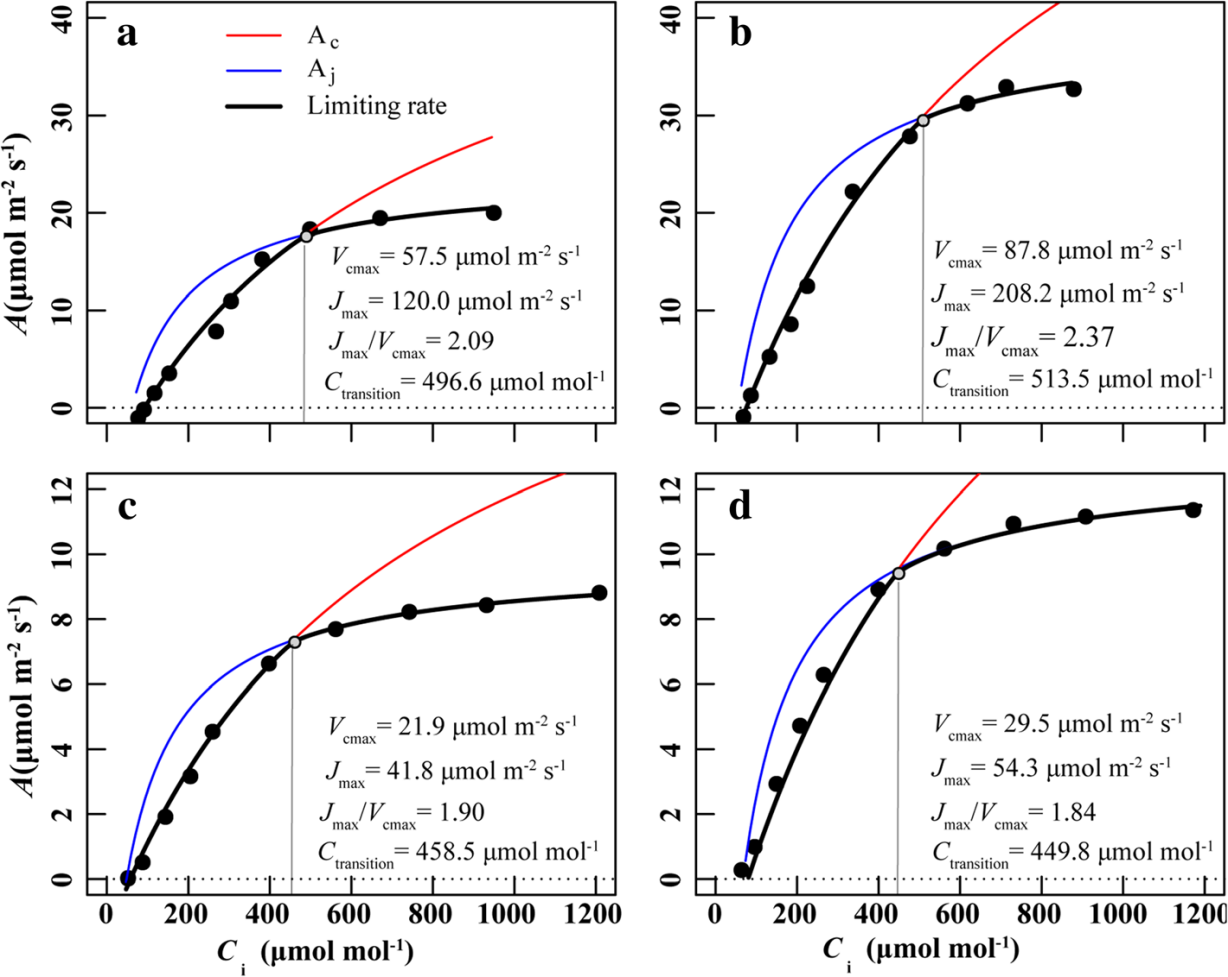
Illustration showing the shift in the limiting step of CO_2_ assimilation. **a** and **b** are limitations of leaves under K-deficiency (−K) and sufficient K supply (+K) treatments, while **c** and **d** are corresponding traits of siliques. CO_2_ assimilation rate limited by RuBP carboxylation (*A*_c_: red line), RuBP regeneration (*A*_r_: blue line), the maximum rate of RuBP carboxylation (*V*_cmax_), the maximum rate of electron transport (*J*_max_) and the intercellular CO_2_ concentration at which the transition from Rubisco to RuBP regeneration limitation occurs (*C*_transition_) were modeled and calculated using the Farquhar, von Caemmerer and Berry (FVCB) photosynthesis model with R *plantecophys* package

The most common purpose of *A*-*C*_i_ curves is to assess in vivo maximum rates of electron transport (*J*_max_) and the maximum rate of RuBP carboxylation (*V*_cmax_), as well as the transformation of RuBP carboxylation and regeneration limitations, which can be altered by the balance of *J*_max_ and *V*_cmax_ and the intercellular CO_2_ concentration (*C*_i_) [25, 26]. In the current study, the *V*_cmax_ and *J*_max_ values of leaves were 2.63–2.98 and 2.87–3.83 times that of siliques, respectively, leading to a lower *J*_max_/*V*_cmax_ in siliques (Fig. 6). The down-regulated *J*_max_/*V*_cmax_ in siliques may be correlated with a greater limitation by RuBP regeneration than by carboxylation, which implies that the weakened electron transport could not meet the requirements for carbon assimilation [26]. The average intercellular CO_2_ concentration at which the transition from Rubisco to RuBP regeneration (*C*_transition_) of leaves was 505.1 μmol CO_2_ mol^−1^, which was 51.0 μmol CO_2_ mol^−1^ higher than that of siliques (Fig. 6). This provided support for our inference, showing that siliques were more restricted in terms of *J*_max_ rather than *V*_cmax_. Further evidence was provided by our investigation of the differences between the estimated and modeled values on the basis of constant *J*_max_/Chl and *V*_cmax_/Chl in leaves and siliques. The estimated-*J*_max_ of siliques were 28.0% and 48.3% lower than modeled-*J*_max_ in the -K and +K treatment, respectively, while the gap between estimated-*V*_cmax_ and modeled-*V*_cmax_ was much smaller (Fig. 5b and Additional file 2: Table S1). It can also be speculated from Fig. 5 that the contributions of *J*_max_ and *V*_cmax_ to the decreased rate of silique photosynthesis were higher than that of α.

Several lines of supporting evidence were provided by comparisons of cell arrangement and chloroplast ultrastructure of the two organs in cross-section. Silique walls had broader cells, however, the cells were fewer in in number (Fig. 4c, d), resulting in a lessened cell surface area per unit of mesophyll volume, and decreasing the light harvesting efficiency of the tissue [27]. In addition, the lack of spongy tissue and larger intercellular airspace had an adverse effect on light scattering [27, 28], thus further decreasing light absorption by siliques. As for the ultrastructure, silique wall chloroplasts were much smaller in size, with a decrease in the number of layers per granum (Fig. 4o-p). This organization of grana has been approved to influence the formation of arrays of PSII-LHCII supercomplexes, ultimately affecting light harvesting [29], and also influencing light-to-charge conversion and electron transport [30]. Nevertheless, the case of discrepancy between comparable actual photochemical efficiency of PSII (Y(II)), maximum quantum yield of PSII (*F*_v_/*F*_m_) and in vivo chlorophyll fluorescence of photosystem II-based electron transport rate (ETR) (Additional file 3: Figure S2), with considerably different *A* and *J*_max_ in the two organs is intriguing. This phenomenon may be accounted for the distribution of electrons to an alternative electron sink such as photorespiration, which may ultimately reduce the pool of electrons available for carboxylation [4]. Another noteworthy fact was that electrons were also consumed by the refixation of internal-cavity respiratory CO_2_ (0.5 to 2.5% v/v in the silique cavity); however, it was not possible to evaluated this by gas exchange or chlorophyll fluorescence [11, 31, 32], as has been proposed for tomato and mango fruit [11, 33]. Nevertheless, it is not known whether these two possibilities exist in silique or which is the main restraint for electrons distribution to CO_2_ assimilation. Additionally, the variation of substrate CO_2_ in chloroplasts will lead to changes in *A* and the pools of Calvin cycle intermediates, which can affect the activity of Rubisco and the capacity for RuBP regeneration [34]. Even with extremely declined *g*_s_, the intercellular CO_2_ concentration (*C*_i_) of siliques was slightly enhanced, possibly caused by restricted CO_2_ diffusion in mesophyll layers or in efficient use of CO_2_. To date, there is no effective method to evaluate the resistance of CO_2_ diffusion through cross-sections of non-leaf organs, especially those with unevenly distributed chloroplasts [35]. Since mesophyll conductance (*g*_m_) is highly dependent on the anatomical traits of mesophyll cells and chloroplasts [36], the micro- or ultra-structures may offer several lines of evidence. Compared with leaves, silique walls had less intercellular airspace and fewer and smaller chloroplasts, which might ultimately decrease gas-phase conductance and the chloroplast area exposed to airspace (*S*_c_/*S*) and, in turn, down-regulated *g*_m_.

### Effects of potassium on leaf and silique photosynthesis

In the present study, notable inhibition of the growth of leaves and siliques was observed under K-depletion. Both organs exhibited the same morphological response to K-deficiency, displaying a reduction in photosynthetic area, leaf (silique) mass per area (*M*_A_) and thickness, which is largely in accordance with previous observations [10, 37]. However, leaf growth was more affected by K-deficiency, with an extremely reduced K status. In higher plants, K is particularly concentrated in growing and reproductive organs, reflecting its ease of transportation [38]. Siliques, which are propagative organs, contain a naturally high K concentration, which is approximately 2-folder greater than that of leaves. Based on studies of the relationship between the biochemical properties of organs (e.g. photosynthesis), it can be hypothesized that the critical K concentration of siliques is higher than that of leaves (1.07%) [16].

In comparison with siliques, leaf photosynthesis was more restricted by K deficiency, which to a certain extent, was attributed to a sensitive stoma, a greatly increased CO_2_ diffusion distance between the chloroplast and cell wall (*D*_chl-cw_), and a down-regulation of *J*_max_ and *V*_cmax_ (Table 2 and Fig. 1). In accordance with previous reports, K-deficiency decreased stomatal length, width and pore area in both organs [14, 16], however, had no influence on stomatal density. The stomatal function, often referred to stomatal conductance to CO_2_ (*g*_sc_), was down-regulated under K-starved conditions in the present study, which is consistent with observations in cotton [39], hickory [13], eucalyptus [14] and sunflower [12]. Therefore, the resistance to CO_2_ diffusion from the atmosphere to the leaf interior was extremely enhanced under conditions of K-deprivation, and this resistance was more pronounced in siliques. However, the higher *C*_i_ values in K-starved leaves versus that of +K treatment indicated that the major influence of K on leaf photosynthesis under current condition may be attributed to lower *g*_m_ and the capacity of CO_2_-fixiation (biochemical activities), rather than stomatal limitations [13]. The *C*_i_ value of silique was independent of K nutrition, which ultimately led to an uncertain causality between main limiting factors and down-regulated *A*.

Leaf *g*_m_ plays an important role in determining CO_2_ acquisition of chlorenchymas [36, 40]. Our previous study indicated that K-deficiency reduced leaf *g*_m_ by decreasing intercellular air spaces, *S*_c_/*S* and enlarging the resistance of the cytoplasm (i.e. *D*_chl-cw_ increased) [10]. K-deficiency significantly increased cytoplasmic resistance of siliques by enhancing *D*_chl-cw_. Additionally, starch accumulation in silique chloroplasts under K-deficiency possibly enlarges chloroplast volume, resulting in enhanced stomatal resistance [18, 41]. Accordingly, silique *g*_m_ may be influenced by K supplies through anatomical variations. In addition to CO_2_ diffusion resistance, severe biochemical limitations on CO_2_ utilization may occur in K-starved organs by down-regulating *J*_max_ and *V*_cmax_ [13, 16]. As already noted, leaves were more sensitive to K starvation with larger discrepancies in *J*_max_ and *V*_cmax_ between the -K and +K treatment versus that of siliques. Furthermore, K-deficiency is involved in the down-regulation of α and Y(II), and an attendant increase of energy dissipation (Y(NPQ)) in K-starved leaves and siliques, which is regarded as an efficient strategy to reduce photodamage [19]. Overall, these results collectively suggest that K plays a crucial role in regulating leaf and silique photosynthesis through its influence on CO_2_ diffusion and biochemical limitations, with siliques exhibiting greater tolerance to K deficiency.

## Conclusions

The present study demonstrated that the CO_2_ assimilation capacity of siliques was much weaker than that of leaves (only account for 35.0%). It can be speculated that this difference is due to decreased function of photosynthetic apparatus, especially the integrated limitations of biochemical processes (*J*_max_ and *V*_cmax_) and α. In comparison with leaves, siliques contained larger but fewer stomata, tightly packed cross-section with larger cells and fewer intercellular air spaces, fewer and smaller chloroplasts with thin grana lamellae. These anatomical traits might be linked to the reduced light capture and CO_2_ diffusion. K-deficiency profoundly decreased leaf and silique photosynthesis by down-regulating *g*_s_, α, Y(II), CE, *V*_cmax_ and *J*_max_. Under K-starvation conditions, the most obvious anatomical features were the swollen chloroplasts with ubiquitous starch grains in silique cells but slightly ambiguous and irregularly arranged granum in leaf cells. Between two contrasting organs, siliques were more less vulnerable to K-depletion, showing a lower decline in K concentration, *g*_s_, *V*_cmax_, *J*_max_, and CO_2_ diffusion resistance in the cytosol. Taken together, these results contribute to an understanding of silique photosynthesis and its response to K-deficiency.

## Methods

### Study site and growth conditions

This study was conducted during the 2014–2015 oilseed rape growing season on a K fertilization experiment located at Wuxue County, Hubei Province, central China (30° 06′46″N, 115° 36′9″E). The location has a subtropical monsoon climate with mean whole-season and wintertide temperatures (from December 2014 to February 2015) of 12.2 and 6.5 °C, respectively, and mean whole-season and wintertide precipitation of 670.0 mm and 222.4 mm. The soil was a sandy loam with the following characteristics in the topsoil layer (0–20 cm): pH 5.7, organic matter 37.1 g kg^−1^, total N 2.0 g kg^−1^, NH_4_OAc-K 45.3 mg kg^−1^, Olsen-P 14.6 mg kg^−1^ and hot-water soluble B 0.82 mg kg^−1^. According to the abundance and deficiency indices of soil-available K [23], the soil type is defined as K-deficient, which would cause yield reduction without the addition of K fertilizer.

### Experimental design

The experiment was carried out in a complete randomized block design with two K treatments and four replicates. The treatments were: (1) sufficient K supply (+K), with a rate of 120 kg K_2_O ha^−1^ (recommended for this region) [42]. (2) K deficiency (−K), with no K fertilizer applied throughout the growing season.

Apart from K, plants received 180 kg N ha^−1^, 90 kg P_2_O_5_ ha^−1^, and 1.6 kg B ha^−1^. Nitrogen (urea, 46% N) was applied in three splits: 60% prior to transplanting, i.e., BBCH (Biologische Bundesantalt, Bundessortenamt and Chemische Industrie) 15–16 [43], 20% at the over-wintering stage (i.e., BBCH 29), and 20% at the start of stem elongation (i.e., BBCH 30). In addition, all P (superphosphate, 12% P_2_O_5_), K (potassium chloride, 60% K_2_O), and B (borax, 10.8%) fertilizers were applied manually as basal fertilizers. The experimental field was plowed and leveled with a rotary tiller, and basal fertilizers were incorporated during the process. The plot measured 20 m^2^, with a length of 10 m and a width of 2 m.

Rapeseed seedlings were grown from seed (Huayouza No.9) in a nursery for 4 weeks and planted by hand at five-leaf stage (i.e., BBCH 15–16, 3–4 g dry weight plant^−1^) on 22 October 2014 in double rows spaced approximately 0.3 m apart, with 0.2–0.3 m between plants, corresponding to 112,500 plants ha^−1^. The oilseed rape was grown under rain-fed conditions. Weeds, pests and disease stresses were controlled by spraying herbicides, insecticides and fungicides according to the local habits so that no obvious weeds, insect pests, and diseases infestations occurred during the cropping season.

### Leaf and silique tagging

In each plot, 40 uniform plants were tagged on 12 November 2014 (3 weeks after transplanting, i.e. BBCH 17), and halved for leaf and silique determination. Twenty leaves (corresponding to 20 plants) were tagged immediately after emergence (length approximately 1.5 cm), and subjected to destructive and non-destructive analyses 20 days later (i.e., BBCH 19) at the point of maximum leaf photosynthesis [44, 45]. The rest of the plant were left until the start of flowering (i.e., BBCH 60–61). For each plant, two adjacent flower buds on the main raceme and opening during the same day were tagged; and approximately 5 days later they had developed into siliques. Siliques were used in experiments after 20 days of growth, when the maximum silique wall area and photosynthetic rate were reached [46].

### Gas exchange

Tagged leaves and siliques were used for gas exchange measurements with a portable, open circuit, infrared gas analysis system (Li-6400, Li-Cor Inc., Lincoln, NE, USA). For each plant, one tagged leaf and two siliques were placed into a Standard Chamber equipped with a 6400–02 LED light source and a 6400-22 L Lighted Conifer Chamber equipped with a 6400–18 RGB light source. Net photosynthesis (*A*) was analyzed for four tagged plants in each treatment in the late morning (10:00–14:00) at a saturating photosynthetic photon flux density (PPFD) of 1500 μmol m^−2^ s^−1^ (90% red light and 10% blue light). The maintained under the following standardized conditions: CO_2_ concentration, 400 μmol mol^−1^ air; flow rate, 500 μmol s^−1^; temperature, 25 ± 0.2 °C; and relative humidity, 50–60%. After equilibration to a steady-state, *A*, stomatal conductance (*g*_s_), and intercellular CO_2_ concentration (*C*_i_) were recorded. Recording data of siliques were corrected for the area because the chamber was not completely filled. Siliques were then halved into two valves (i.e. silique walls) along the replum, the valves were then flattened and mounted on black cardboard after removing the seeds and scanned digitally together with a green reference card (25 cm^2^) using an Epson ES-1200C scanner (Epson, Long Beach, CA, USA). The silique area was determined using Image-Pro Plus 4.5 software (National Institutes of Health, Bethesda, Maryland).

Light- and CO_2_-response curves were constructed for three leaves and six siliques (each replicate consisted of two siliques) which had been previously acclimated to saturating light conditions for 20 min. For light-response curves, gas exchange was determined at 11 levels of PPFD, (2000, 1800, 1500, 1200, 1000, 800, 500, 200, 100, 50, 0 μmol m^−2^ s^−1^). Meanwhile, the *C*_a_ was maintained at 400 μmol mol^−1^ air. For CO_2_-response curves, the *C*_a_ in the chamber was adjusted across a series of concentrations (400, 300, 200, 100, 50, 400, 600, 800, 1000, 1200, and 1500 μmol CO_2_ mol^−1^) at a constant PPFD of 1500 μmol m^−2^ s^−1^. The temperature and relative humidity during analysis of light and CO_2_ responses were uniformly controlled at 25 ± 0.2 °C and 50–60%. In all cases, the parameters were recorded and the areas were corrected (for siliques only) after the gas exchange rate stabilized at the given *C*_a_ or PPFD. Apparent quantum yield (α) and carboxylation efficiency (CE) were represented by the initial slope of the linear part of the light-response curve (0 ≤ PPFD ≤200 μmol m^−2^ s^−1^) and the CO_2_-response curve (0 ≤ *C*_i_ ≤ 200 μmol CO_2_ mol^−1^).

According to Farquhar, von Caemmerer and Berry (1980; FVCB) photosynthesis model, the net photosynthetic rate is limited mainly by Rubisco carboxylation or by RuBP regeneration [25]. The intercellular CO_2_ concentration at which the transition from Rubisco to RuBP regeneration limitation occurs was calculated in R3.3.1 (R core Team, 2016) using *plantecophys* package [47]. The maximum rate of RuBP carboxylation (*V*_cmax_) and the maximum rate of RuBP regeneration (*J*_max_) were also evaluated using the same R package.

### Chlorophyll fluorescence imaging

Imaging of chlorophyll fluorescence parameters was performed immediately after gas exchange measurements using a MINI-Version Imaging-PAM (IMAG-MIN/B, Walz, Effeltrich, Germany), which can be used to assess image areas up to 2.4 × 3.2 cm. The instrument employs a bank of blue LEDs (peak wavelength 470 nm) and a 1/3″ CCD camera (640 × 480 pixels). Before experiments, intact leaves and siliques were adapted to the dark for at least 30 min. Immediately before measurement, three leaves and siliques were excised with a razor blade and put into black bags to avoid light reflections. Images of the minimal fluorescence yield of dark-acclimated samples (*F*_o_) were acquired at low frequencies of pulse-modulated measuring light, and the maximal fluorescence yield (*F*_m_) was measured with an 800 ms saturation pulse. Samples were then exposed to actinic illumination, and images of the steady-state chlorophyll fluorescence (*F*_t_) were captured. Subsequently, the maximal fluorescence yield under light (*F*_m_^′^) was measured during exposure to saturation pulse. The maximum quantum yield of PSII (*F*_v_/*F*_m_), the effective quantum efficiency of PSII (Y(II)) and the quantum yield of light-induced non-photochemical fluorescence quenching (Y(NPQ)) were calculated with Imaging-Win software (Walz, Effeltrich, Germany).

### Morpho-physiological traits

Six leaves and twelve siliques per treatment were used to determine areas according to the aforementioned approach. Leaves and silique walls were oven dried to constant weight at 60 °C, and dry mass per area (*M*_A_) was calculated by dividing the weight of the dry matter by the area. The samples were then milled, and subsamples of 0.15 g were digested with H_2_SO_4_-H_2_O_2_ [48], before K concentration determination using a flame 321 photometer (M-410, Cole-Parmer, Chicago, IL, USA). Thereafter, another three leaves and six siliques (with seeds and septa removed) were cut into small segments (approximately 5 mm). After extraction with 80% (*v*/v) alcohol for 24 h, chlorophyll concentration was determined using a UV–vis spectrophotometer (UV2102, Unico, China) after extracting with 80% (v/v) alcohol for 24 h [49].

### Anatomical analysis

Cross-sections of the leaves and siliques used for gas exchange measurements were prepared. Segments (approximately 1 × 1 mm) obtained from intercostal areas of fresh leaves and from the middle valves of siliques were fixed in 2.5% glutaraldehyde (*v*/v) in 0.1 M phosphate buffer (pH 7.2) for 4 h at 4 °C. Subsequently, the segments were post-fixed with 1% osmium tetroxide for 1 h at 25 °C. The samples were further dehydrated in a graded ethanol series, and embedded in Spurr’s epoxy resin, before polymerization.

For the light microscope observation, samples were cut into 1 μm transverse sections using a LKB-5 ultramicrotome 359 (LKB Co., Ltd., Uppsala, Sweden), and stained with 0.5% toluidine blue. Micrographs were captured at a magnification of 100× using a Nikon Eclipse E600 microscope equipped with a Nikon 5 MP digital microscope camera DS-Fi1 (Nikon Corporation, Kyoto, Japan). Four samples were analyzed per treatment for both leaves or siliques. For each sample, thickness of at least four cross-sections was measured. *M*_A_ is the product of leaf thickness and density [50], therefore leaf density was estimated by dividing *M*_A_ by thickness.

For the ultrastructural observations, ultrathin sections (90 nm) were examined using a transmission electron 360 microscope (H-7650, Hitachi, Japan) after staining with 2.5% uranyl acetate (*w*/*v*) and lead citrate. The numbers of chloroplast in the leaf spongy tissue cells and mesocarp cells (*n* ≥ 30) were counted under the magnification of 5000×. The corresponding chloroplast length (*L*_chl_) and thickness (*T*_chl_), and the chloroplast distance from the cell wall (*D*_chl-cw_) were measured for at least 30 randomly selected chloroplasts at a magnification of 25,000–30,000×. Chloroplast surface area (*S*_chl_) and volume (*V*_chl_) were calculated (assuming that the chloroplasts were ellipsoids) according to the Cesaro formula:

1 $$ {S}_{\mathrm{chl}}=4\times \pi {\times}^3\sqrt{{\left(d\times {e}^2\right)}^2} $$

2 $$ {V}_{\mathrm{chl}}=\frac{4}{3}\times \pi \times d\times {e}^2 $$

Where *d* = 0.5 × *L*_chl_; and *e* = 0.5 × *T*_chl_. The average distance of chloroplasts from the cell wall (*D*_chl-cw_, n ≥ 30) was determined according to the method described by Tomás et al. (2013) [36].

For stomatal trait determination, the leaf and silique segments (5 × 5 mm) were fixed in 2.5% glutaraldehyde (v/v) at 4 °C for 2 h. Segments were then washed twice in 0.1 M phosphate buffer (pH 7.2) and, followed by dehydrated in a graded ethanol series. After further drying and spraying with gold, the treated segments were observed and photographed with a scanning electron microscope (JSM-5310LV, Jeol Co, Tokyo, Japan). The numbers of stomata in the lower epidermis and exocarp were counted at a magnification of 500×, and the stomatal frequency (*n* ≥ 20) was calculated by dividing the stomata number by the area of the field of view. In addition, at least 50 randomly selected stomatas were selected to measure the length (*L*_stomata_) and width (*W*_stomata_) at a magnification of 3500×. Assuming the stomatas were ellipsoids, the single stomatal pore area (A_stomata_) would be given as:

3 $$ {A}_{\mathrm{stomata}}=\frac{1}{4}\times \pi \times {L}_{\mathrm{stomata}}\times {W}_{\mathrm{stomata}} $$

Therefore, the total stomatal pore area was the product of A_stomata_ and stomata frequency.

### Statistical analyses

Descriptive statistical analyses were used for the measured parameters to obtain means and standard error (SE). All data were subjected to two-way analysis of variance (ANOVA) with SPSS 18.0 software (SPSS, Chicago, IL, USA). The differences between mean values were compared with Duncan’s multiple range test; *P* < 0.05 was considered to indicate statistical significance. Graphics were prepared using the ORIGINPRO 8.5 software (OriginLab Corporation, Northampton, MA, USA).

## Additional files

Additional file 1: Illustration showing development progress of *Brassica napus* L. (PDF 434 kb)

Additional file 2: The gap between estimated and modeled (theoretical) values of silique under K deficiency (−K) and K sufficient (+K) conditions. (PDF 282 kb)

Additional file 3: The relationship between chlorophyll fluorescence of photosystem II-based electron transport rate (ETR) at saturating photosynthetic photon flux density (PPFD) of 1500 μmol m^−2^ s^−1^ with LI-6400 XT equipped with an integrated leaf chamber fluorometer (LI-6400-40) and maximum rate of electron transport (*J*_max_) estimated from *A*-*C*_i_ curve. (PDF 411 kb)

## Abbreviations

*A*: Net photosynthetic rate
*C*_a_: Ambient CO_2_ concentration
CE: Carboxylation efficiency
Chl: Chloroplast concentration
*C*_i_: Intercellular CO_2_ concentration
*D*_chl-cw_: the distance between chloroplast and cell wall
*F*_o_: the minimal fluorescence yield of dark-acclimated samples
*F*_v_/*F*_m_: the maximum quantum yield of PSII
*g*_m_: Mesophyll conductance
*g*_s_: Stomatal conductance
*J*_max_: the maximum rate of electron transport
K: Potassium
LAI: Leaf area index
*M*_A_: Dry mass per area
PAI: Pod area index
PPFD: Photosynthetic photon flux density
*S*_chl_: Chloroplast surface area
*V*_chl_: Chloroplast volume
*V*_cmax_: the maximum rate of RuBP carboxylation
Y(II): Actual photochemical efficiency of PSII
Y(NPQ): the quantum yield of light-induced non-photochemical fluorescence quenching
α: Apparent quantum yield.
