# Supplementary figures and images for: The photosynthetic and structural differences between leaves and siliques of Brassica napus exposed to potassium deficiency
Source: BMC Plant Biol. 2017 Dec 11;17:240. doi: 10.1186/s12870-017-1201-5 (PMC5725657; doi:10.1186/s12870-017-1201-5)

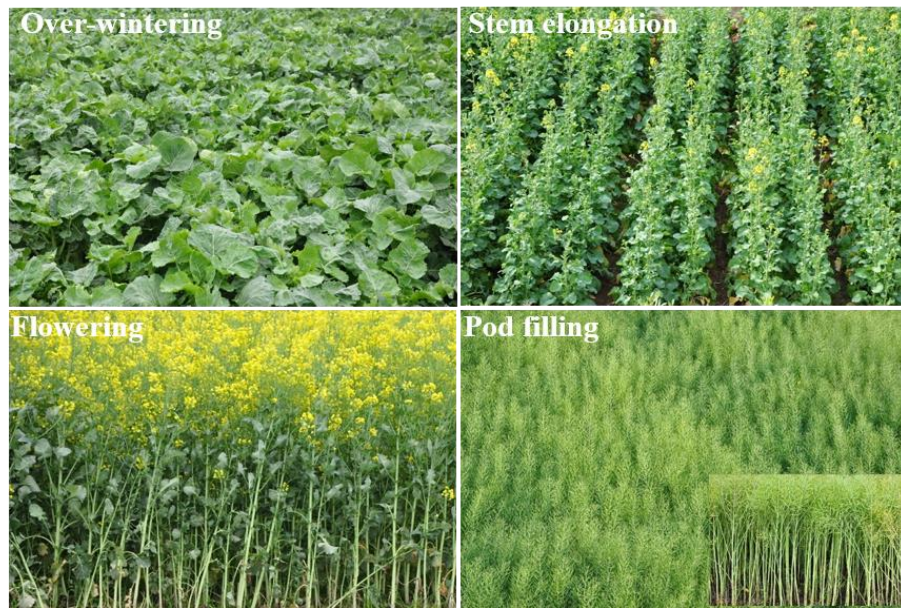

**Figure S1** Illustration showing development progress of *Brassica napus* L.

Supplement: Supplementary file 1 — Illustration showing development progress of Brassica napus L. (PDF 434 kb) [file 12870_2017_1201_MOESM1_ESM.pdf]
