# Supplementary material for: The photosynthetic and structural differences between leaves and siliques of Brassica napus exposed to potassium deficiency
Source: BMC Plant Biol. 2017 Dec 11;17:240. doi: 10.1186/s12870-017-1201-5 (PMC5725657; doi:10.1186/s12870-017-1201-5)
Supplement: Supplementary file 2 — The gap between estimated and modeled (theoretical) values of silique under K deficiency (−K) and K sufficient (+K) conditions. (PDF 282 kb) [file 12870_2017_1201_MOESM2_ESM.pdf]

Table S2 The gap between estimated and modeled (theoretical) values of silique under K deficiency (-K) and K sufficient (+K) conditions. The percentages listed below were calculated from Figure 5 as the relative change of estimated α, CE, J_{max} and V_{cmax} when serving modeled values as control. The positive and negative values indicate that the estimated values were higher or lower than modeled values.

Treatment	α	CE	J_{max}	V_{cmax}
-K	-16.6%	5.7%	-28.0%	-21.3%
+K	-23.2%	1.4%	-48.3%	-33.5%
