# Supplementary material for: The photosynthetic and structural differences between leaves and siliques of Brassica napus exposed to potassium deficiency
Source: BMC Plant Biol. 2017 Dec 11;17:240. doi: 10.1186/s12870-017-1201-5 (PMC5725657; doi:10.1186/s12870-017-1201-5)
Supplement: Supplementary file 3 — The relationship between chlorophyll fluorescence of photosystem II-based electron transport rate (ETR) at saturating photosynthetic photon flux density (PPFD) of 1500 μmol m−2 s−1 with LI-6400 XT equipped with an integrated leaf chamber fluorometer (LI-6400-40) and maximum rate of electron transport (J max) estimated from A-C i curve. (PDF 411 kb) [file 12870_2017_1201_MOESM3_ESM.pdf]

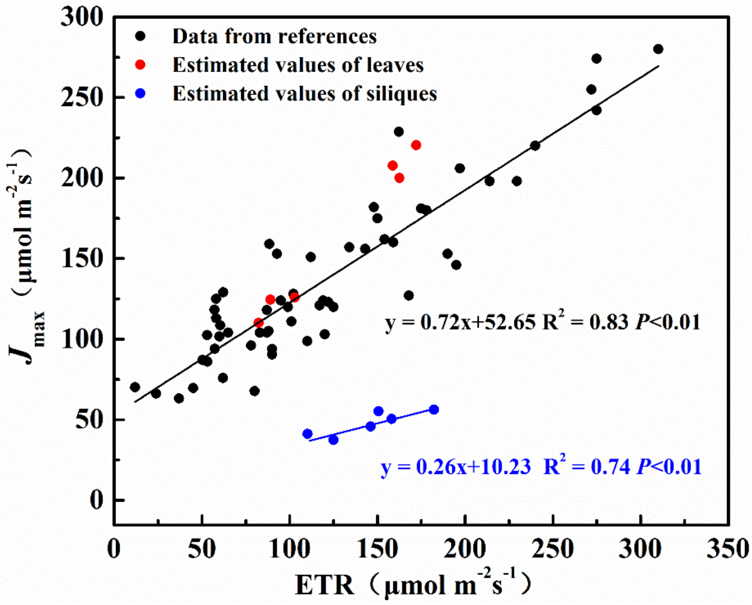

Figure S2 The relationship between chlorophyll fluorescence of photosystem II-based electron transport rate (ETR) at saturating photosynthetic photon flux density (PPFD) of 1500 μmol $m^{-2}s^{-1}$ with LI-6400 XT equipped with an integrated leaf chamber fluorometer (LI-6400-40) and maximum rate of electron transport (J_{max}) estimated from A-C_i curve. The red and blue circles indicate estimated ETR of leaf and silique, while the black circles denote the data compiled from the following references: Flexas et al. (2007); Zhang et al. (2007); Araujo et al. (2008); Yang et al. (2008); Zhang and Wen (2008); Ribeiro et al. (2009); Robredo (2010); Stavang et al., 2010; Cano et al. (2011); Rosenthal et al. (2010); Sun et al. (2011); Dahal et al., 2012.

References:

1. Flexas J, Ortuño MF, Ribas-Carbo M, Diaz-Espejo A, Flórez-Sarasa ID, Medrano H. Mesophyll conductance to CO_2 in *Arabidopsis thaliana*. New Phytol. 2007;175:501-511.
2. Zhang SB, Hu H, Xu K, Li ZR, Yang YP. Flexible and reversible responses to different irradiance levels during photosynthetic acclimation of *Cypripedium guttatum*. J Plant Physiol. 2007;164:611-620.
3. Araujo WL, Dias PC, Moraes GABK, Celin EF, Cunha RL, Barros RS, Damatta FM. Limitation to photosynthesis in coffee leaves from different canopy positions. Plant Physiol Bioch. 2008;46:884-890.
4. Yang WJ, Chen HF, Zhu FY, Hu MQ, Jiang DA. Low concentration of bisulfite enhances photosynthesis in tea tree by promoting carboxylation efficiency in leaves. Photosynthetic.

2008;46:615-617.

5. Zhang LL, Wen DZ. Photosynthesis, chlorophyll fluorescence, and antioxidant enzyme responses of invasive weed *Mikania micrantha* to *Bemisia tobaci* infestation. Photosynthetic. 2008;46:457-462.
6. Ribeiro RV, Machado EC, Santos MG, Oliveira RF. Seasonal and diurnal changes in photosynthetic limitation of young sweet orange trees. Environ Exp Bot. 2009;66:203-211.
7. Robredo A, Pérez-López U, Lacuesta M, Mena-Petite A, Muñoz-Rueda A. Influence of water stress on photosynthetic characteristics in barley plants under ambient and elevated CO_2 concentration. Biol Plantarum. 2010;54:285-292.
8. Stavang JA, Pettersen RI, Wendell M, Solhaug KA, Junttila O, Moe R, Olsen JE. Thermoperiodic growth control by gibberellin does not involve changes in photosynthetic or respiratory capacities in pea. J Exp Bot. 2010;61:1015-1029.
9. Cano FJ, Sánchez-Gómez D, Gascó A, Rodriguez-Calcerrada J, Gil L, Warren CR, Aranda I. Light acclimation at the end of the growing season in two broadleaved oak species. Photosynthetic. 2011;49:581-592.
10. Rosenthal DM, Locke AM, Khozaei M, Raines CA, Long SP, Ort DR. Over-expressing the C_3 photosynthesis cycle enzyme Sedoheptulose-1-7 Bisphosphatase improves photosynthetic carbon gain and yield under fully open air CO_2 fumigation (FACE). BMC Plant Biol. 2011;11:123.
11. Sun JD, Zhang JS, Larue CT, Huber SC. Decrease in leaf sucrose synthesis leads to increased leaf starch turnover and decreased RuBP regeneration-limited photosynthesis but not Rubisco-limited photosynthesis in *Arabidopsis* null mutants of *SPSA1*. Plant Cell Environ. 2011;34:592-604.
12. Dahal K, Gadapati W, Savitch LV, Singh J, Hüner NPA. Cold acclimation and *BnCBF17*-over-expression enhance photosynthetic performance and energy conversion efficiency during long-term growth of *Brassica napus* under elevated CO_2 conditions. Planta. 2012;236:1639-1652.
